# A Preliminary Study of I-Gel: A New Supraglottic Airway Device

**Published:** 2009-02

**Authors:** Ashish Kannaujia, Uma Srivastava, Namita Saraswat, Abhijeet Mishra, Aditya Kumar, Surekha Saxena

**Affiliations:** 1 Lecturer, Department of Anaesthesiology & Critical Care, S N. Medical College, Agra; 2 Professor, Department of Anaesthesiology & Critical Care, S N. Medical College, Agra; 3 PG Students, Department of Anaesthesiology & Critical Care, S N. Medical College, Agra; 4 PG Students, Department of Anaesthesiology & Critical Care, S N. Medical College, Agra; 5 Professor, Department of Anaesthesiology & Critical Care, S N. Medical College, Agra; 6 Professor, Department of Anaesthesiology & Critical Care, S N. Medical College, Agra

**Keywords:** I-gel, Supraglottic airway device, Non–inflatable mask, Oropharyngeal seal pressure

## Abstract

**Summary:**

This preliminary study on I-gel™ (Intersurgical Ltd, Wokingham, U K) was conducted on 50 consecutive patients of ASA physical status I-III, to determine the ease of insertion, time to achieve effective airway, oropharyngeal seal pressure and airway stability on head and neck movement.

After premedication with midazolam and fentanyl, induction was done with propofol and I-gel was inserted according to manufacturer's instruction. An effective airway was confirmed by bilateral chest movement, square wave on capnograph and SpO_2_>95%.

The success rate at first attempt was 90% with a median insertion time of 11 sec (range 8-45sec). Five patients (10%) needed second attempt while none needed 3^rd^ attempt. The manipulation needed to achieve effective airway were increasing the depth of insertion of I-gel in 4 (8%) cases, jaw thrust or chin lift in 2(4%) cases. Oropharyngeal seal pressure was 20 cm of H_2_O (16-40 cm of H_2_O). Gastric tube placement was done in 50% of the cases; it was easy and successful in all the cases. No significant adverse event was noted in any of the patient in perioperative period.

Our initial experience showed that I-gel is a simple, easy to use supraglottic airway device with a high success rate at first time insertion.

## Introduction

**Fig 1:**
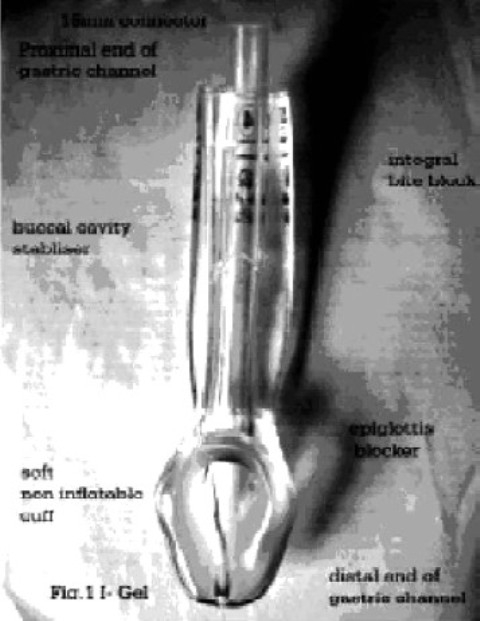
I Gel

I-gel™ (Intersurgical Ltd, Wokingham, U.K.) is a new supraglottic airway device with anatomically designed, non inflatable mask, which is soft, gel like and transparent, made of thermoplastic elastomer^1^. The soft, non inflatable cuff fits snugly onto the perilaryngeal framework and its tip lies in the proximal opening of the oesophagus, isolating the oropharyngeal opening from the laryngealinlet. The outer cuff shape ensures that the blood flow to the laryngeal and perilaryngeal framework is maintained and helps the possibility to reduce neurovascular compression trauma to the nerves. The device has buccal cavity stabilizer which has propensity to adopt its shape to oropharyngeal curvature of the patients. It is an atomically widened and concaved to eliminate the potential for rotation, thereby reducing the risk of malposition. This buccalcavity stabilizer houses airway tubing and separate gastric channel. The tube section is firmer than the soft bowl of the gastric channel. The firmness of tube section and its natural oropharyngeal curvature allows the device to be inserted by grasping the proximal end of it against the hard palate into the pharynx without inserting the fingers into the mouth of the patients^1^. The smooth contiguous surface of the device from the tip of the bowl to the proximal end of the tube, allows the device to easily slide posteriorly along the hard palate, pharynx and hypopharynx. The device has integral bite block which is marked with a horizontally placed black line, which acts as a guide to depth of insertion. The device also has a channel for gastric tube drain (except size 1), which runs through the device from its proximal opening at the slide of flat connector wing to the distal tip of the non inflatable mask. The gastric channel allows suction, detection of leak and passage of gastric tube. The maximum size of gastric tube which can be passed through different sizes is given in Table 1. The device also has an epiglottic blocker (Fig. 1) which prevents downfolding of the epiglottis and obstruction of the distal opening airway.

**Table 1:** Recommended size of I-gel, maximum size of gastric tube and endotracheal tube which can be inserted through it.

Size of I-gel	Bodyweight (kg)	Maximumsize of gastric tube	Maximum size of ETT which can be passed
1	1-5	N/A	3.5
1.5	5-12	10	4.5
2	10-25	12	5.0
2.5	25-35	12	6.0
3	30-60	12	6.0
4	50-90	12	7.0
5	90+	14	8.0

The size of I-gel is selected according to patient's body weight (Table 1). I-gel can be used for intubation as a conduit. The maximum size of endotracheal tube which can be passed through the definite size I-gel is given in Table 1.

## Methods

Fifty consecutive patients of either sex belonging to ASA physical status I – III were recruited for this study after approval from institutional ethical committee with informed written consent. The patients with anticipated difficult airway, pregnancy, obesity and those who required surgery in positions other than supine or lithotomy position or required IPPV were excluded from the study.

All the patients received oral alprazolam and ranitidine in the night before surgery and in the morning on the day of surgery. In the operation room an IV line was secured and monitors were applied which included ECG, pulseoximetry and capnography. Induction of anaesthesia in each case was done in supine position with the head on the standard pillow (7-10 cm). Patients were given, fentanyl (1-1.5μg.kg^−1^) and midazolam (0.02 mg.kg^−1^ body weight). After preoxygenation for 3 minutes, each patient received induction dose of propofol (2-2.5 mg.kg^−1^) over 30-40 seconds with end point of induction being loss of eyelash reflex. Face mask ventilation was done with 33% O_2_ in N_2_O and 1-2% of halothane until optimal conditions for I-gel insertion were attained (Jaw relaxation, no movement). Additional increments of propofol were given as and when required until level of anaesthesia adequate for insertion of device was achieved. The insertion of I-gel was done according to manufacturer's instruction. The front, back and sides of the cuff were lubricated with water based jelly. The patient's head was placed in ‘sniffing the morning air’ position. The lubricated device was grasped along the integral bite block and was introduced into the mouth in the direction towards the hard palate and was glided downwards and backwards along the hard palate until definite resistance was felt. The device was connected to breathing circuit and patientventilated manually. An effective airway was confirmed by bilateral symmetrical chest movement, square waveform on capnograph and normal SpO_2_ (>95%). The device was secured with adhesive tape.

If the airway was not effective, manipulations were done in the form of increasing the depth of insertion, giving jaw thrust or chin lift or changing size of the device. If it was not possible to maintain an effective airway after 3 insertion attempts; the device was taken as failure and alternative technique (proseal-LMA or ETT) was used.

Ventilation of patient was manually assisted until the spontaneous breathing resumed. Oropharyngeal seal pressure was determined by closing the expiratory valve at a fixed gas flow of 5 L/min (Magill circuit) and recording the airway pressure at which the gas leaked into the mouth^2^.

Anaesthesia was maintained on O_2_, N_2_O (66%) and halothane (1-2%) with spontaneous ventilation.

Towards the end of the procedure but before discontinuing anaesthetic, the stability of the device was evaluated in different head/neck positions. This involved placing the head and neck in four sequential positions-head on standard pillow, head rotated to side, chin lift, and head without standard pillow and recording five consecutive tidal volumes under a constant level of anesthesia depth^3^. At the end of procedure all the patients breathed 100% O_2_ during emergence from anaesthesia. The device was removed when patient was able to open the mouth on command. The patient was inspected about any injury to the lips, teeth or tongue and device was inspected for any blood stain.

## Results

The patient characteristics and type of surgeries are shown in Table 2.

**Table 2:** Patient's characteristics and type of surgeries

Variables	
**Age (yrs)-range(median)**	20 – 75 (42)
**Weight (kg)-range(median)**	42 – 90 (56)
**GenderM/F(number)**	22/28
**ASA Physical status/II/III**	35/9/6
**Type of Surgeries**	
** Gynecology**	24
** General surgery**	18
** Orthopaedics**	6
**Urology**	2
**Duration of Anaesthesia - Median**	45min
**Range**	30-60min

Data are shown as number of patients(median)

Size 3 was used in 84% of cases and size 4 was used in 16%. In 2 patients the device was replaced with larger size to achieve better seal.

The device was easy to insert and remove. The success rate at first attempt was 45/50 (90%) with a median insertion time of 11 seconds (range 8-45 seconds). Five patients needed 2^nd^ attempt while none needed 3^rd^ attempt or had failure of insertion. The most common manipulations to achieve effective airway was increasing the depth of insertion of I-gel (8%). Airway manipulations in the form of chin lift or jaw thrust were needed in 2% patients (Table 3). In two cases the device was replaced with a larger size to achieve better seal.

**Table 3:** Airway management details

**Size 3/4(n)**	42/8
**Insertion Attempts 1/2/3/Failed(n)**	45/5/0/0
**Time For Insertion -**	
** Median**	11 sec
** Range**	8-45 sec
**Easyremoval(n)**	50
**Airway Manipulation Required(n)**	5
** Jaw Thrust(n)**	1
** Chin Lift(n)**	0
** Increasing the depth of insertion**	4
**Changing the Size of Device**	2
**Oropharyngeal seal Pressure (cm of H_2_O)**	20 (range 16-40)
**Attempts at gastric tube insertion 1/2/3/Failed**	20/0/0/0

Data are shown as number of patients

Oropharyngeal leak pressure was 20 cm of water (range 16-40).

Gastric tube placement was done only in 50% of the patients and it was easy and successful in each case. No patient had gastric distention. There was no clinical evidence of aspiration in any patient.

No significant adverse event was noted in any patient. The incidence of adverse events during perioperative period was low (Table 4). Two patients (4%) had suboptimal oxygenation (SpO_2_ <95%) for which airway manipulation in the form of increasing the depth of insertion was done which rectified the problem. None of the patient suffered hypoxia (SpO_2_ <90%). During emergence 2 patients (4%) had cough and 2 (4%) patient complained of sore throat in the postoperative period which subsided within 24 hrs. None of the patient complained of dysphonia or dysphagia.

**Table 4:** Adverse events

	N	%
**Suboptimal oxygenation(SpO_2_<95%)**	2	4
**Hypoxia (SpO_2_<90%)**	0	0
**Coughing**	2	4
**Laryngospasm**	0	0
**Leak**	0	0
**Gastric insufflation**	0	0
**Hiccups**	0	0
**Regurgitation**	0	0
**Aspiration**	0	0
**Injuryto Lip, Teeth, Gum**	0	0
**Blood on Device**	0	0
**Sore Throat**	2	4
**Dysphagia**	0	0
**Dysphonia**	0	0

Data are shown as number of patients and percentage.

## Discussion

The results of the present clinical trial have shown many advantages of I-gel. These include high success rate at first attempt, easy insertion and shorter time to achieve effective airway. The additional advantages are high seal pressure and stability of device despite changes in position of head and neck. All the anaesthetists stated that the placement of I-gel was certainly easier than any other currently available supraglottic device. This greater stability is primarily related to the anatomical design of the non-inflatable cuff. The ridge at the proximal end of mask catches the base of tongue thus prevents the device from moving and so contributes to the positional stability of the device after placement^4^. Since no cuff inflation is needed in this device, there is shorter time to achieve effective airway.

A supraglottic airway device without inflatable cuff has some potential advantages including easier insertion and minimal risk of tissue compression^5^–^7^ whereas supraglottic device with inflatable cuff can absorb anesthetic gases leading to increased mucosal pressure^8^. I-gel may find a place during CPR due to high success rateat first attempt along with quick insertion time^9^,^10^. Also, easy ventilation of chest without air leak during chest compression may have additional advantage.

Intra operative problems like arterial desaturation and haemodynamic changes were not seen inany patient probably due to shorter time for successful placement of the device.

As this is a new device very little published data is available regarding its use during anaesthesia. Most of the studies are manikin or cadaver studies and these are mainly aimed to evaluate the ease of success of insertion by non-anaesthetist. I-gel has been found to have easiest insertion in various types of manikins^11^. Levitan and Kinkle^4^ studied the positioning and mechanics of this device in 65 non embalmed cadavers with endoscopies and neck dissections. Aglottic opening score (POGO) of >50% was obtained in all 65 cases. In each of the neck dissections the bowl of device was found covering the laryngeal inlet. In a study on cadavers, I-Gel was consistently positioned over laryngeal inlet as confirmed by endoscopy, radiography and dissection^4^. It was also used to facilitate endotracheal intubation^12^.

We concluded that I-gel is a simple, excellent and easy to use supraglottic airway device. It is easy to insert without need of many manipulations with maintenance of airway in a short time. The device is very effective and useful for adult patients requiring surgical procedures of 30-60 minutes duration while breathing spontaneously. We strongly believe that I-gel may become very popular due to its superior qualities of speedy yet successful insertion and ventilation. However more studies with large number of patients are required to further validate our results before recommending its widespread use. Our study was conducted on spontaneously breathing anaesthetized patients, we didnot studied it for controlled ventilation.
